# The effect of task order predictability in audio-visual dual task performance: Just a central capacity limitation?

**DOI:** 10.3389/fnint.2012.00075

**Published:** 2012-09-11

**Authors:** Thomas Töllner, Tilo Strobach, Torsten Schubert, Hermann J. Müller

**Affiliations:** ^1^Department of Psychology, Ludwig-Maximilians-Universität MünchenMunich, Germany; ^2^Department of Psychology, Humboldt Universität BerlinBerlin, Germany; ^3^Department of Psychological Sciences, Birkbeck College LondonLondon, UK

**Keywords:** attention, decision making, executive control, central bottleneck, PCN, LRP

## Abstract

In classic Psychological-Refractory-Period (PRP) dual-task paradigms, decreasing stimulus onset asynchronies (SOA) between the two tasks typically lead to increasing reaction times (RT) to the second task and, when task order is non-predictable, to prolonged RTs to the first task. Traditionally, both RT effects have been advocated to originate exclusively from the dynamics of a central bottleneck. By focusing on two specific electroencephalographic brain responses directly linkable to perceptual or motor processing stages, respectively, the present study aimed to provide a more detailed picture as to the origin(s) of these behavioral PRP effects. In particular, we employed 2-alternative forced-choice (2AFC) tasks requiring participants to identify the pitch of a tone (high versus low) in the auditory, and the orientation of a target object (vertical versus horizontal) in the visual, task, with task order being either predictable or non-predictable. Our findings show that task order predictability (TOP) and inter-task SOA interactively determine the speed of (visual) perceptual processes (as indexed by the PCN timing) for both the first and the second task. By contrast, motor response execution times (as indexed by the LRP timing) are influenced independently by TOP for the first, and SOA for the second, task. Overall, this set of findings complements classical as well as advanced versions of the central bottleneck model by providing electrophysiological evidence for modulations of both perceptual and motor processing dynamics that, in summation with central capacity limitations, give rise to the behavioral PRP outcome.

## Introduction

In classic Psychological-Refractory-Period (PRP) dual-task paradigms, the time taken to respond to the stimulus of the second task typically increases with decreasing inter-task interval (i.e., stimulus onset asynchrony, SOA), whereas there is no influence of inter-task SOA on reaction times (RT) to the stimulus of the first task (e.g., Welford, 1952; Pashler and Johnston, 1989). This well-established and extensively studied effect has traditionally been explained in terms of a sequential processing model consisting of three stages: (1) a *perceptual* stage, which selects the task-relevant stimulus (e.g., based on a spatial characteristic: left versus right positioning of the stimulus relative to the vertical midline of the display) and, if required, extracts the response-critical stimulus attribute (e.g., exact featural identity: red versus green) required for subsequent response decisions; (2) a *central* stage which decides upon the appropriate motor response (e.g., left versus right index finger press) on the basis of a pre-specified task setting (i.e., stimulus-response, S-R, and mapping); and (3) a *motor* stage, which produces and executes this response. While both perceptual and motor stages are generally assumed to operate in parallel, the commonly advocated view (e.g., Pashler, 1984; Luck, 1998; Schubert, 1999) is that the effect of inter-task SOA on RTs to the second task may originate exclusively from a processing *bottleneck* located at the central stage, in particular: central-stage processing of the second task is delayed until central processing of the first task has been completed. Accordingly, RTs to the second task depend on the onset of responses to the first task, rather than the onset of the respective (first-task) stimuli.

Recent findings (e.g., Schubert, 1996, 2008; Jiang et al., 2004; Sigman and Dehaene, 2006), however, have challenged the traditional view that responses to the *first* task are processed independently of the inter-task interval. For instance, responses to the first task are slowed down compared to when this task is executed in isolation (e.g., Jiang et al., 2004; Sigman and Dehaene, 2006), or when the sequence of the two upcoming (dual) tasks is made unpredictable (e.g., De Jong, 1995; Szameitat et al., 2002, 2006; Sigman and Dehaene, 2006). To explain this set of findings, Sigman and Dehaene (2006) introduced an “*extended central bottleneck*” *view*, according to which additional central executive processes, including task scheduling and task disengagement (see also Lien et al., 2003; Liepelt et al., 2011; Strobach et al., 2012, in press), are assumed to give rise to the increased processing times for the first task especially at short SOAs (e.g., <300 ms). Crucially, both task control processes are scheduled within the *central* system (involving the operation of executive control); thus, again assuming solely *central processes* as origin of the RT cost associated with unpredictable, relative to predictable, task orders.

On this background, the aim of the present electroencephalogram (EEG) study was twofold: First, we intended to gain deeper insights into the question of whether the SOA effect on RTs to the first task under conditions of unpredictable task order is indeed due to the dynamics of task coordination processes that operate exclusively at the *central stage*, as proposed by the “extended central bottleneck” model of Sigman and Dehaene (2006; see also Schubert, 1996); or, alternatively, whether there might also be modulations evident at the preceding *perceptual* and/or the subsequent *motor* stage that, when combined with the central processing dynamics, contribute to this RT effect. Second, we asked whether the SOA effect on RTs to the second task is, again, solely driven by central capacity limitations, as advocated by traditional central bottleneck models (e.g., Pashler, 1994), and/or whether this effect may be further influenced by the predictability of the task order. To address these questions, we employed a 2-alternative forced-choice (2AFC) audio-visual dual task, requiring participants to identify the pitch of a tone (high versus low) in the auditory, and the orientation of a laterally presented target object (vertical versus horizontal) in the visual, task, with the order of the dual tasks being either fixed (predictive task order) or random (non-predictive task order), with variable inter-task intervals (SOAs). In addition, we combined mental chronometry data with two specific electroencephalographic brain responses directly linkable to either pure perceptual or pure motor stages of the information-processing stream.

The first EEG parameter, the *Lateralized-Readiness-Potential* (LRP), is a well-known and extensively studied event-related potential (ERP) component generally agreed to reflect the activation and execution of effector-specific motor responses (e.g., Coles, 1989; Osman and Moore, 1993; Eimer, 1998). In more detail, the LRP is negativity strongest over the motor areas contralateral to the side of a uni-manual response, typically elicited in the 150 ms time window pre-response. To dissociate the LRP from overlapping motor response-unspecific ERPs, the waveforms recorded ipsilateral to the response side are subtracted from contralateral waveforms, resulting in the so-called (contralateral-minus-ipsilateral) LRP difference wave. These subtractions can be performed time-locked to either stimulus or response onset. Accordingly, the timing of the stimulus-locked LRP (sLRP) can be regarded as indexing the start of effector-specific motor activation after the completion of response selection (i.e., central) processes (e.g., Sommer et al., 2001; Töllner et al., 2011b), whereas the time demands required by response execution processes are derivable from the response-locked LRP (rLRP) onset timing (e.g., Miller, 2007).

The second parameter of interest, the *Posterior-Contralateral-Negativity* (PCN), is a similarly prominent and extensively explored electroencephalographic brain response that has been linked to the focal-attentional selection of task-relevant target objects in visual space (e.g., Luck and Hillyard, 1994; Eimer, 1996; Woodman and Luck, 1999). [Traditionally, this component has been referred to as N2-posterior-contralateral (N2pc). However, based on recent evidence (e.g., Shedden and Nordgaard, 2001) that underscores the independence of this component in terms of both timing and activation from the non-lateralized N2, we prefer the term PCN (instead of N2pc) in order to avoid misleading associations or interpretations.] Specifically, the PCN is a negative-going deflection most prominent over the visual areas contralateral to the side of an attended object, elicited—depending on a variety of *top-down* (e.g., Eimer and Kiss, 2008; Töllner et al., 2010, 2012a) and *bottom-up* (e.g., Brisson et al., 2007; Töllner et al., 2011a) factors—in the time window approximately 175–300 ms post-stimulus. As for LRP computations, it is strongly recommended to subtract the waveforms recorded ipsilateral to the stimulus side from contralateral waveforms to cancel out overlapping target selection-unspecific ERPs.

**Figure 1:**
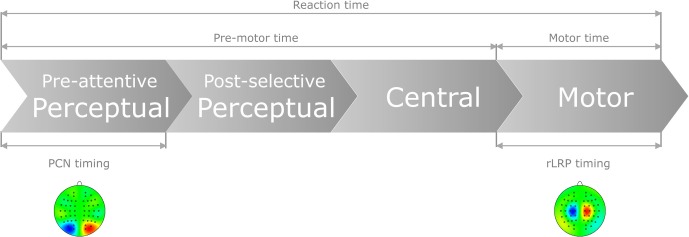
**Schematic of the present approach to control for pure perceptual and/or pure motor capacity limitations as a function of task order predictability (TOP) and stimulus onset asynchrony (SOA) in dual task.** In particular, by computing the difference waves of the PCN and LRP responses, the following time demands can be electro-cortically extracted: (1) Pre-attentive perceptual processes, as determined by feature-contrast and salience coding, necessary to focally select the visual target stimulus; (2) Motor production processes, as determined by the required motor effector, necessary to activate and execute the selected response.

Taken together, for auditory and visual responses, the coupling of mental chronometry to the rLRP allows us to dissociate pre-motor (i.e., perceptual and central processes) and motor processes that, in combination, may contribute to the interactive RT effect of “task-order predictability” and “stimulus-onset-asynchrony” in audio-visual dual-task performance (see Figure 1). In addition, for responses to visual stimuli, we can further split pre-motor times into processing components related to pre-attentive, perceptual and, respectively, post-selective, perceptual plus central (i.e., stimulus-response translation) processes on the basis of PCN computations.

## Materials and methods

### Participants

Thirteen participants (seven female) took part in the present study. Their ages ranged from 24 to 32 (median 28) years. All had normal or corrected-to-normal vision and reported no history of neurological disorders. Observers were either paid or received course credit for participating. One observer was excluded due to excessive eye movement artifacts. The experimental procedure was approved by the ethics committee of the Department of Psychology, Ludwig-Maximilians-University Munich, in accordance with the Code of Ethics of the World Medical Association (Declaration of Helsinki).

### Stimuli and study design

Visual stimulation consisted of two colored shape stimuli (radius: 1.2° of visual angle) presented against a black background and positioned equidistantly (visual angle: 3.0°) from a white central fixation cross in the lower visual field. On each trial, one of the two lateralized locations contained a task-relevant target stimulus, equally likely defined in the pre-instructed color *red* (CIE 0.544, 0.403, 68) or *blue* (CIE 0.213, 0.264, 68), together with a task-irrelevant distracter stimulus at the opposite location defined in the alternative color (blue or red, respectively). Each stimulus outline contained a grating composed of three black bars (0.4° × 2.4°) separated by two gaps (0.3° × 2.4°), which were randomly oriented either vertically or horizontally. Auditory stimuli were pure sine-waves tones, of a frequency of either 350 or 900 Hz, and of 100 ms duration.

The experiment was performed in a dimly lit, sound-attenuated, and electrically shielded experimental cabin (Industrial Acoustics Company GmbH). Visual stimuli were presented on a 17″ computer screen, mounted at a viewing distance of approximately 75 cm. Auditory stimuli were presented simultaneously via two stereo loudspeakers, placed approximately 10 cm to the left and right side of the monitor, respectively. In order to obtain a reasonable number of trials necessary to analyze all experimental conditions of interest (see below), two separate recording sessions were conducted for each individual participant, with the second session performed within one week of, and at a similar time of day, as the first session. One experimental session consisted of 24 blocks of 48 trials each, resulting in a total of 2304 trials for each participant for both sessions. Each session was further divided into four parts of six blocks each, with two parts with predictive and two with non-predictive task-order. The sequence of these four parts was counterbalanced across subjects, but held constant across the two sessions for each individual participant. A trial started with the presentation of a white fixation cross for 500 ms, which was immediately followed by the stimuli of the first task (i.e., visual or auditory, respectively) presented for 100 ms. After a randomly chosen SOA of 150, 300, or 600 ms, the stimuli of the second task (i.e., visual or auditory, respectively) appeared for 100 ms. Trials were terminated by the participant's response(s) or after a maximum response window of 3 s for both tasks. In case of an incorrect response, the word “FEHLER” (German word for “ERROR”) was centrally presented for 1 s, signaling erroneous behavior. Subsequently, a blank screen was shown during an intertrial interval of 1 s. Participants were clearly instructed to maintain central eye fixation throughout the experiment and to respond as quickly and accurately as possible, with the order of motor responses matching the order of (visual versus auditory) task occurrence.

In both tasks, there were always two stimuli concurrently presented at two lateralized locations. In the auditory task, both loudspeakers presented one-and-the-same stimulus requiring participants to identify the pitch of a tone (i.e., high versus low). The visual task, by contrast, involved the presentation of two different stimuli so as to be able to compute the PCN component (see also Brisson and Jolicoeur, 2007a,b). Prior to the start of each experiment, the task-relevant (visual) stimulus was specified by a semantic pre-cue (e.g., the word “BLUE”) indicating the defining color of the target stimulus (in the example: blue) in the upcoming block of trials. Independent of the target-defining color, however, participants task was to identify the target's orientation (i.e., vertical versus horizontal). It should be noted that this difference between the two types of task, which permitted the PCN to be computed for visual stimuli, had no consequences for the second parameter of interest: the LRP, which is computed relative to the respective side of the executed motor response (see above). Further, we deliberately introduced the same task requirements for both the auditory and the visual task (i.e., stimulus identification, rather than detection or localization; see Töllner et al., 2012b, for a systematic comparison of different task settings), in order to obtain comparable response latencies (see RT analysis below). Participants responded, for example, to the auditory task with a single key press with the left hand, using the index and middle finger to indicate the high versus low pitch of the tones, respectively; and with a single key press using the right index and middle finger to indicate the target's (vertical versus horizontal) orientation in the visual task. The S–R mappings were reversed across hands and fingers after the first half of each experimental session and counterbalanced across participants. Prior to the start of the first as well as second half of each experimental session, at least one block of practice was administered to permit participants to become familiar with the required S–R mapping in each task. After each block, participants received summary performance statistics (mean error rate and RT).

### EEG recording and data analysis

The EEG was continuously digitized from 64 Ag/AgCl active electrodes (actiCAP system, BrainProducts Munich) at 1 KHz. Electrodes were mounted on an elastic cap (Easy Cap, FMS) and placed in accord to the International 10–10 System (American Electroencephalographic Society, 1994). The horizontal and vertical electrooculogram was monitored by means of electrodes placed at the outer canthi of the eyes, and the superior and inferior orbits, respectively. All electrophysiological signals were amplified by BrainAmp amplifiers (BrainProducts, Munich) using a 0.1–250 Hz bandpass filter, and filtered offline with a 0.5–40 Hz band-pass (Butterworth infinite-impulse-response filter, 24 dB/Oct). All electrodes were referenced to FCz and re-referenced offline to averaged mastoids. Impedances were kept below 5 kΩ.

Prior to segmenting the EEGs, the raw data was visually inspected in order to identify and manually remove non-stereotypical noise in the signals. This was followed by an infomax independent-component analysis (ICA) run to identify components representing blinks and/or horizontal eye movements, and to remove these artefacts before back-projection of the residual components. The continuous EEG was then epoched into 3.0 s segments, ranging from 1.3 s before to 1.7 s after stimulus onset. Next, a baseline correction was performed based on the 200 ms pre-stimulus interval. Only trials with correct responses in both (dual) tasks and without artifacts—defined as any signal exceeding ±60 μV, bursts of electromyographic activity (permitted maximal voltage steps/sample point of 50 μV), and activity fluctuating less than 0.5 μV within 500 ms (indicating “dead” channels)—were considered on an individual-channel basis for further analysis. The signals were then re-epoched into 0.6 s segments ranging from 200 ms before to 400 ms s after stimulus onset for the PCN analysis and, respectively, into 1.2 s segments ranging from 1 s before to 200 ms after response onset for the rLRP analysis, before the ERP waveforms were averaged.

The LRP was quantified by subtracting ERPs measured at medial central electrodes (C3/C4) ipsilateral to the response side from contralateral ERPs. The onset latencies of the LRPs were computed according to the jackknife-based scoring method (Ulrich and Miller, 2001), which defines the LRP onset as the point in time at which the LRP activation meets a specific criterion value relative to the pre-stimulus baseline. As proposed by Ulrich and Miller (2001), we used 90% of the maximum LRP activation as optimal criterion for defining rLRP onset latencies. LRP amplitudes were calculated averaging five data points before and after the maximum deflection obtained in the 250 ms pre-response time interval. The PCN was computed by subtracting ERPs measured at lateral parieto-occipital electrode sites (PO7/PO8) ipsilateral to the target's location from contralateral ERPs. The latencies of the PCNs were defined individually as the maximum negative-going deflection in the 150–350 ms post-stimulus interval. PCN amplitudes were computed averaging five data points before and after this maximum deflection.

For both the first and second (dual) task responses, differences in behavioral (RTs, error rates) as well as electrophysiological measures (rLRP onset latencies/amplitudes; PCN latencies/amplitudes) were assessed by carrying out separate two-way repeated-measure analyses of variance (ANOVAs) with the factors Task Order Predictability (TOP) (predictive, non-predictive) and SOA (150 ms, 300 ms, and 600 ms). Significant main effects and/or interactions were further examined by means of *post-hoc* comparisons (Tukey HSD).

## Results

### Responses to the first task

#### Behavior

**Figure 2:**
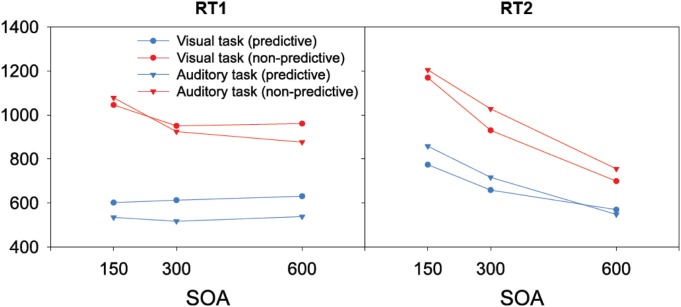
**Reaction times of the present study as a function of task order (first task, second task), task order predictability (predictive, non-predictive), and stimulus onset asynchronies (150 ms, 300 ms, 600 ms)**.

When the auditory task was performed first, we found RTs to be modulated interactively by TOP and SOA. As can be seen from the left panel of Figure 2, RTs increased monotonically with decreasing inter-task interval for non-predictive task orders (denoted by red lines), whereas there was no SOA effect for predictive task orders (denoted by blue lines). Statistically, both main effects [TOP: *F*_(1, 11)_ = 45.09, *p* < 0.001; SOA: *F*_(2, 22)_ = 26.44, *p* < 0.001] as well as their interaction [*F*_(2, 22)_ = 20.732, *p* < 0.001] were significant. *Post-hoc* analyses confirmed that, for non-predictive task orders, RTs increased from long to intermediate SOAs [*p* < 0.05] and from intermediate to short SOAs [*p* < 0.001]. By contrast, no statistical differences were evident among the various SOA levels for predictive task order conditions [all *p* > 0.07]. With regard to the error rates (depicted in Table 1), more incorrect responses were made when the order of the two tasks was non-predictive (2.5% vs. 1.4%) and when they were separated by short (3.0%) rather than intermediate (1.5%) and long (1.4%) inter-task intervals, yielding significant main effects of TOP [*F*_(1, 11)_ = 12.24, *p* < 0.001] and SOA [*F*_(2, 22)_ = 12.00, *p* < 0.001].

**Table 1:** **Error rates in the present study as a function of task order (first task, second task), task order predictability (predictive, non-predictive), and stimulus onset asynchrony (150 ms, 300 ms, 600 ms)**.

**Task**	**SOA**	**First task**		**Second task**	
		**predictive**	**non-predictive**	**predictive**	**non-predictive**
Visual	150	2.0	3.0	3.6	4.0
Task	300	1.7	3.1	4.2	3.2
	600	1.7	2.5	4.5	3.5
Auditory	150	4.6	3.5	2.4	3.7
Task	300	3.5	2.6	1.1	2.0
	600	3.5	3.0	0.9	1.9

The same overall data pattern was revealed when the visual task was performed first (left panel of Figure 2). RTs again increased monotonically with decreasing inter-task interval for non-predictive task orders (denoted by red lines), with no SOA influences evident for predictive orders (denoted by blue lines). As for the auditory task, this was substantiated by a significant main effect of TOP [*F*_(1, 11)_ = 49.01, *p* < 0.001], which interacted significantly with SOA [*F*_(2, 22)_ = 5.362, *p* < 0.05]. In more detail, intermediate and short inter-task intervals differed significantly for non-predictive task orders [*p* < 0.01], but not for predictive orders [all *p* > 0.07]. Participants again made more errors with non-predictive relative to predictive task orders (2.9% vs. 1.8%), evidenced by a significant main effect of TOP [*F*_(1, 11)_ = 7.723, *p* < 0.05].

#### Posterior-contralateral-negativity

**Figure 3:**
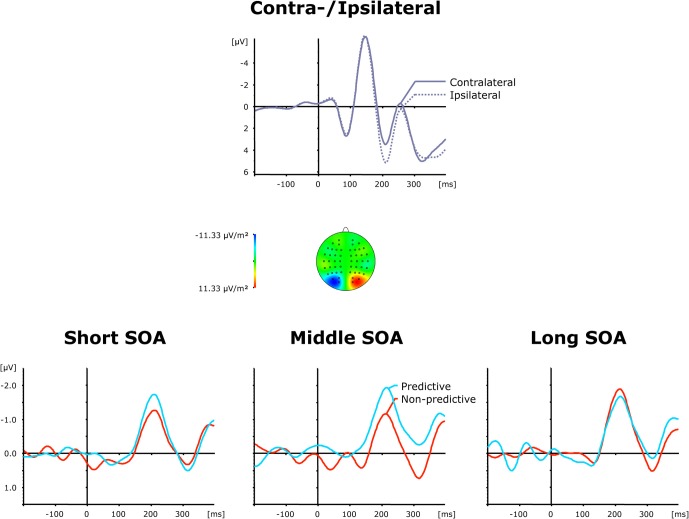
**Grand-averaged event-related brain responses time-locked to the visual stimulus of the first task at electrodes PO7/PO8. Top panel**: Waveforms contra—and ipsilateral to the target location. **Central panel**: Topographical map of the PCN scalp distribution at the point in time when the difference between contra—and ipsilateral waveforms reached its maximum. These maps were computed by mirroring the contra-minus-ipsilateral difference waves to obtain symmetrical values for both hemispheres (using spherical spline interpolation). **Bottom panel**: PCN difference waves obtained by subtracting ipsilateral from contralateral activity for each of the six TOP × SOA conditions.

Grand average ERP waveforms elicited by visual first-task displays are shown separately for contra—and ipsilateral target stimuli with respect to the hemisphere of the recording electrode (PO7/PO8) in the top panel of Figure 3, while the bottom panel presents the corresponding (contralateral-minus-ipsilateral) difference waves as a function of SOA (short, intermediate, and long) and TOP (predictive, non-predictive). For all six (TOP × SOA) conditions, a solid PCN was evoked, visible as a more negative (i.e., less positive) voltage in the time range approximately 150–250 ms *post-stimulus*. To statistically corroborate that the PCN was elicited reliably for the first task, we initially performed a repeated-measures ANOVA with the single factor Period (Baseline versus PCN activation). Baseline activation values were determined—similar to the PCN amplitudes (see above)—by averaging across five sample points prior to and following the maximum negatively directed deflection in the 200 ms pre-stimulus interval. This analysis revealed the effect of Period [*F*_(1, 11)_ = 13.81, *p* < 0.003] to be significant, confirming the presence of the PCN.

As further can be seen from (the bottom panel of) Figure 3, the PCN was more pronounced for predictive relative to non-predictive task orders at short (−1.72 μV vs. −1.29 μV) and intermediate (−2.16 μV vs. −1.06 μV), but not long (−1.83 μV vs. −2.05 μV), inter-task intervals. In addition, for non-predictive task orders, the rise of the PCN appeared to be slightly delayed for short (221 ms) and intermediate (218 ms), as compared to long (209 ms), inter-task intervals; in contrast, this pattern was reversed for predictive task orders (short: 207 ms; intermediate: 211 ms; long: 223 ms). Both observations were substantiated by a significant main effect of TOP for PCN amplitudes [*F*_(1, 11)_ = 6.74, *p* > 0.025], as well as a significant interaction of both factors for PCN amplitudes [*F*_(2, 22)_: 3.99, *p* < 0.033] and latencies [*F*_(2, 22)_ = 6.22, *p* < 0.007]. Subsequent *post-hoc* contrasts confirmed faster PCN elicitation with predictive relative to non-predictive task orders for short inter-task intervals, and vice versa for long intervals (all *p* < 0.05).

#### Lateralized-readiness-potential

**Figure 4:**
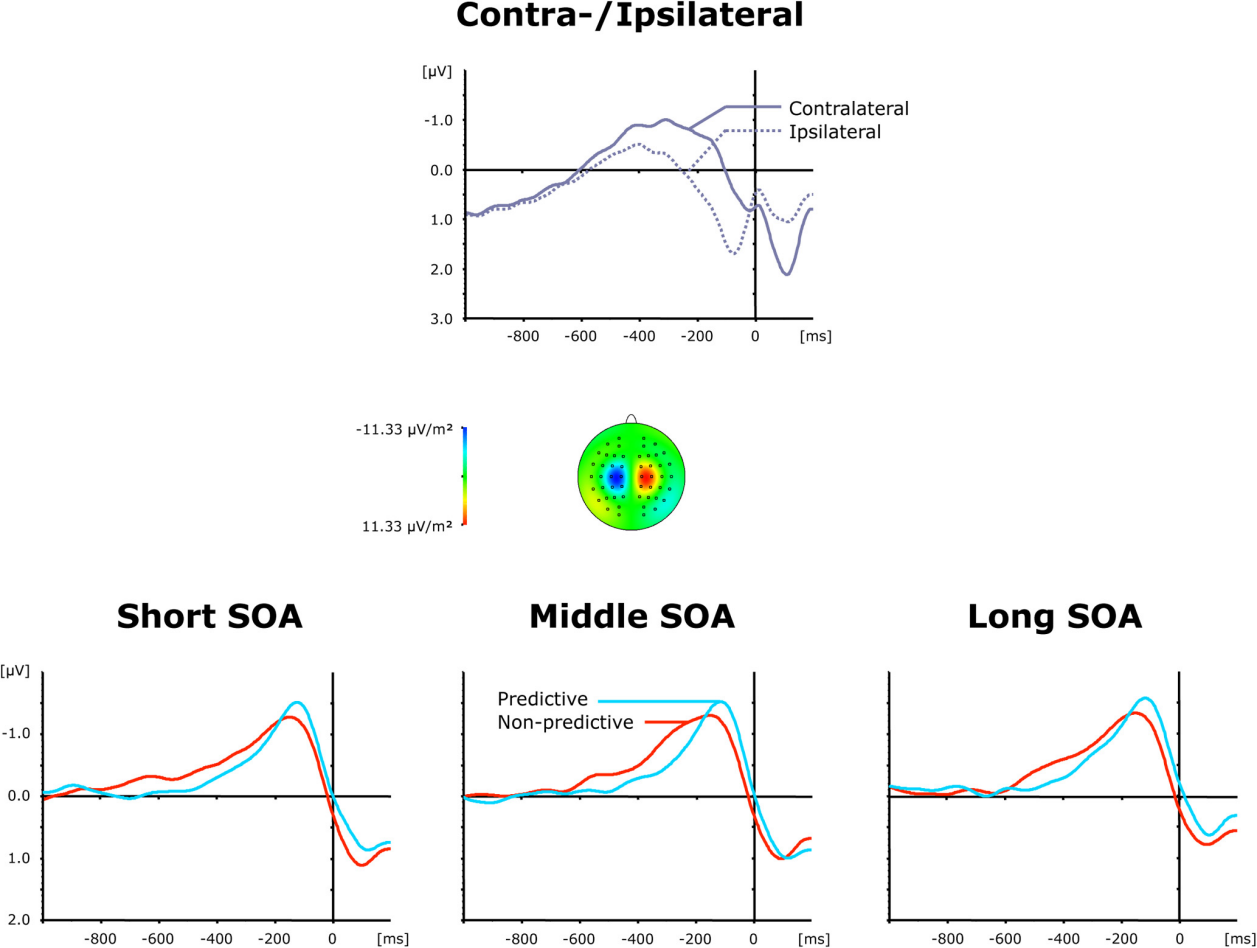
**Grand-averaged event-related brain responses time-locked to the motor response of the first task at electrodes C3/C4. Top panel**: Waveforms contra—and ipsilateral to the response side. **Central panel**: Topographical map of the LRP scalp distribution at the point in time when the difference between contra—and ipsilateral waveforms reached its maximum. These maps were computed by mirroring the contra-minus-ipsilateral difference waves to obtain symmetrical values for both hemispheres (using spherical spline interpolation). **Bottom panel**: LRP difference waves obtained by subtracting ipsilateral from contralateral activity for each of the six TOP × SOA conditions.

The top panel of Figure 4 presents grand average ERP waveforms elicited by both visual and auditory stimuli, separately for the recording electrodes (C3/C4) contra—and ipsilateral to the side of the respective motor response, while the bottom panel shows the corresponding (contralateral-minus-ipsilateral) difference waves as a function of inter-task SOA (short, intermediate, and long) and TOP (predictive, non-predictive). All six (TOP × SOA) conditions triggered a solid LRP, visible as a more negative (i.e., less positive) voltage most pronounced approximately in the 200 ms pre-response time window. First, we compared activation values obtained during the baseline and LRP time windows (see above) in a repeated-measure ANOVA with the factor Period (Baseline versus LRP activation). A highly significant main effect [*F*_(1, 11)_ = 13.01, *p* < 0.004] of Period corroborated that the LRP was reliably triggered. As further illustrated in Figure 4 (bottom panel), the rise of the LRP occurred earlier (relative to response onset) and was more pronounced for predictive (158 ms, −1.32 μV) relative to non-predictive task order trials (210 ms, −1.06 μV), with no differences discernable across inter-task intervals. Statistically, these observations were confirmed for LRP onset latencies [*F*_*c*(1, 11)_: 5.61, *p*_*c*_ > 0.037], but failed to reach significance level for LRP amplitudes [*F*_(1, 11)_: 2.05, *p* > 0.180].

### Responses to the second task

#### Behavior

As illustrated in the right panel of Figure 2, RTs to the *auditory* second task were generally increased with non-predictive (denoted by red lines) relative to predictive (denoted by blue lines) task orders [*F*_(1, 11)_ = 53.40, *p* < 0.001], and decreasing inter-task intervals [*F*_(2, 22)_ = 459.74, *p* < 0.001], with the latter replicating the classic SOA effect in PRP dual-tasks. Further, TOP and SOA interacted significantly [*F*_(2, 22)_ = 19.84, *p* < 0.001], owing to a monotonically increasing TOP effect from long to intermediate SOAs [*p* < 0.001], and intermediate to short SOAs [*p* < 0.001]. In addition, participants exhibited significantly [*F*_(2, 22)_ = 6.86, *p* < 0.01] more errors with short (4.0%) relative to intermediate (3.1%) and long (3.2%) inter-task intervals (see Table 1). There was no main effect of, or interaction with, TOP on errors.

Similar to the response times to the first task, RTs to the *visual* second-task displays generally matched the overall pattern of the auditory RTs: both main effects [TOP: *F*_(1, 11)_ = 36.40, *p* < 0.001; SOA: *F*_(2, 22)_ = 117.11, *p* < 0.001] as well as their interaction [*F*_(2, 22)_ = 44.43, *p* < 0.001] were significant. As can be seen in the right panel of Figure 2, the TOP effect was again stronger for intermediate than for long inter-task intervals [*p* < 0.001], and even more pronounced for short relative to intermediate SOAs [*p* < 0.001]. No effects reached statistical significance in the error data.

#### Posterior-contralateral-negativity

**Figure 5:**
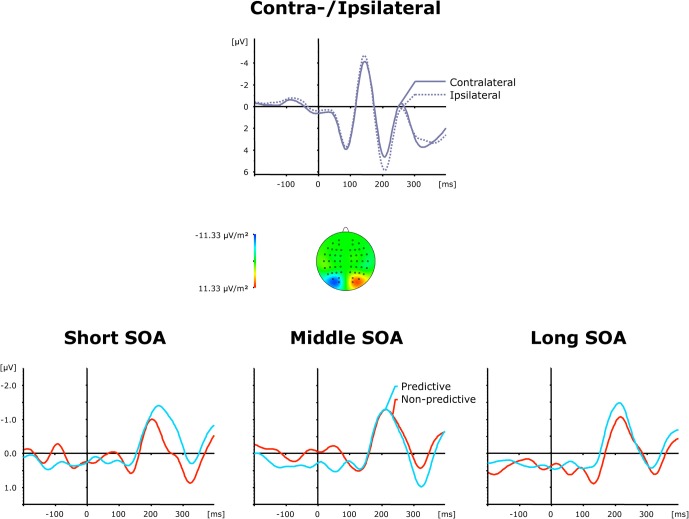
**Grand-averaged event-related brain responses time-locked to the visual stimulus of the second task at electrodes PO7/PO8. Top panel**: Waveforms contra—and ipsilateral to the target location. **Central panel**: Topographical map of the PCN scalp distribution at the point in time when the difference between contra—and ipsilateral waveforms reached its maximum. These maps were computed by mirroring the contra-minus-ipsilateral difference waves to obtain symmetrical values for both hemispheres (using spherical spline interpolation). **Bottom panel**: PCN difference waves obtained by subtracting ipsilateral from contralateral activity for each of the six TOP × SOA conditions.

Grand average ERP waveforms elicited by visual second-task displays are presented separately for contra—and ipsilateral target stimuli relative to the hemisphere of the recording electrode (PO7/PO8) in the top panel of Figure 5; the bottom panel shows the corresponding difference waves as a function of SOA (short, intermediate, and long) and TOP (predictive, non-predictive). In all six experimental conditions, a solid PCN was elicited, evident as a more negative (i.e., less positive) voltage in a time range similar to the PCNs evoked by the first task. An initial ANOVA with the single main term Period (Baseline versus PCN activation) yielded a highly significant main effect [*F*_(1, 11)_ = 10.99, *p* < 0.007], confirming PCN elicitation in response to visual second-task displays. As further shown by the bottom panel of Figure 5, the TOP effect on the PCN timing was dependent on the inter-task interval. The PCN was delayed for non-predictive as compared to predictive task orders at short SOAs (233 ms vs. 204 ms), but expedited at long SOAs (202 ms vs. 214 ms), without any timing difference at intermediate SOAs (210 ms vs. 210 ms)—statistically confirmed by a significant TOP × SOA interaction on PCN latencies [*F*_(2, 22)_: 3.49, *p* < 0.048]. By contrast, there were no reliable differences in the associated PCN amplitudes [main effect TOP: *F* < 1, *p* > 0.416; main effect SOA: *F* < 1, *p* > 0.548; interaction: *F* < 1.15, *p* > 0.338].

#### Lateralized-readiness-potential

**Figure 6:**
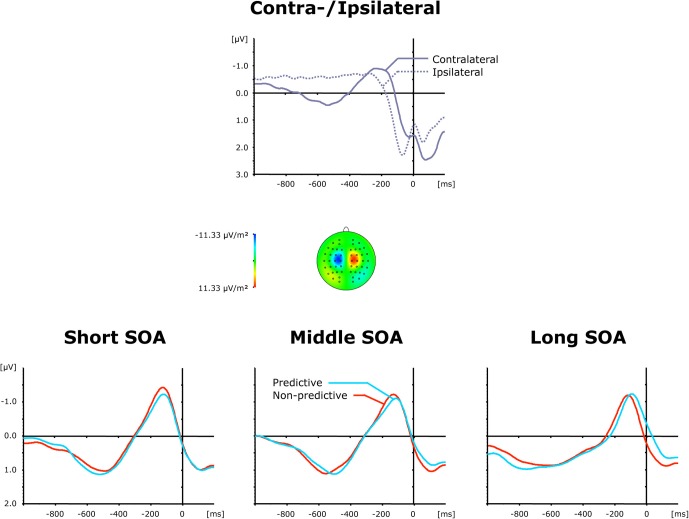
**Grand-averaged event-related brain responses time-locked to the motor response of the second task at electrodes C3/C4. Top panel**: Waveforms contra—and ipsilateral to the response side. **Central panel**: Topographical map of the LRP scalp distribution at the point in time when the difference between contra—and ipsilateral waveforms reached its maximum. These maps were computed by mirroring the contra-minus-ipsilateral difference waves to obtain symmetrical values for both hemispheres (using spherical spline interpolation). **Bottom panel**: LRP difference waves obtained by subtracting ipsilateral from contralateral activity for each of the six TOP × SOA conditions.

The top panel of Figure 6 presents average ERP waveforms elicited by visual and auditory stimuli belonging to the second task, separately for the recording electrode (C3/C4) contra—and ipsilateral to the motor-response side, while the bottom panel shows the corresponding difference waves as a function of SOA (short, intermediate, and long) and TOP (predictive, non-predictive). In all six experimental conditions, a solid LRP was triggered, which can be seen as a more negative (i.e., less positive) voltage strongest in the 200 ms pre-response time range, and following the preceding contralateral positivity (i.e., representing the corresponding contralateral motor response) associated with the first task. Statistically, an initial ANOVA with the single factor Period (Baseline versus LRP activation) confirmed LRP presence [*F*_(1, 11)_ = 11.73, *p* < 0.006]. Furthermore, as shown in Figure 6 (bottom panel), the rise of the LRP occurred faster (relative to response onset) for long (134 ms) relative to intermediate × (162 ms) and short (161 ms) inter-task intervals, with a slightly expedited LRP onset for predictive (126 ms) relative to non-predictive (142 ms) task order trials at long SOAs. By contrast, there were no reliable differences in LRP magnitude across the SOA conditions. Statistically, these observations were substantiated by a significant main effect of SOA for LRP onset latencies [*F*_*c*(2, 22)_: 3.57, *p*_*c*_ > 0.045], and non-significant effects [TOP: *F* < 1, *p* > 0.712; SOA: *F* < 1, *p* > 0.638; interaction: *F* < 1, *p* > 0.879] for LRP amplitudes. Note that the TOP × SOA interaction on LRP onset latencies failed to reach significance [*F*_*c*_ < 1, *p*_*c*_ > 0.794].

## Discussion

When investigating the processing dynamics underlying bottlenecks in PRP-type of dual-task paradigms, two behavioral findings robustly emerge: (1) for the first task, increasing RTs with decreasing inter-task SOAs when the task order is made non-predictive; and (2), independent of TOP, increasing RTs with decreasing inter-task SOAs for the second task. Both RT effects traditionally have been advocated to originate *exclusively* from capacity limitations (i.e., bottlenecks) residing at the *central* stage (i.e., central system) of the information-processing stream (e.g., Pashler, 1994; Sigman and Dehaene, 2006). Here, we provide electrophysiological evidence that challenges this classic view.

### Electroencephalographic measurement of perceptual capacity limitations in dual-task

Evidence for capacity limitations at a perceptual stage of processing derives from the activation pattern of the PCN, which—owing to its latency and extraction method from the ERP—has been demonstrated to reflect pure perceptual processes (e.g., Luck and Hillyard, 1994; Eimer, 1996) within the visual modality. In particular, the latency of this lateralized brain response is indicative of the time required by the human visual system to focally select the target amongst distracter items in visual space (e.g., Woodman and Luck, 1999; Töllner et al., 2011a), whereas its magnitude indicates the amount of focal-attentional resource allocation to the target location.

For the *first* task, we found TOP and inter-task SOA to interactively influence the speed and activation strength of the PCN: for predictive task orders, the PCN was elicited (on average ~14 ms) earlier with short relative to long inter-task intervals, whereas this pattern was reversed for non-predictive task orders, with (on average ~14 ms) shorter PCN latencies for long relative to short SOAs. Associated with this, the magnitude of the PCN was reduced for short and intermediate, but not long, inter-task SOAs when the order of the two upcoming (dual) tasks could not be predicted. At variance with classical PRP models—such as the passive bottleneck model (e.g., Pashler, 1994)—which do not envisage bottlenecks to arise at early sensory processing levels, this set of findings provides support for a recent proposal of Szameitat and colleagues (2006; see also De Jong, 1993), namely, that processes of task-order scheduling may not operate exclusively at a central level of tasks or task sets. Instead, task-order scheduling may already affect perceptual processes via *priming* the modality of the task expected to be presented first. In particular, Szameitat et al. (2006) studied a PRP paradigm in which the order of the two upcoming (dual) tasks changed randomly on a trial-by-trial basis. They found RT (to both the first and the second task) to be speeded markedly when participants had to perform the identical, relative to a different, task order as on the previous trial. As suggested by Szameitat et al., this RT benefit may result, at least in part, from pre-activating the respective sensory modality when participants could properly anticipate the correct task order. However, since their study used functional magnetic resonance imaging in relation to RT data, any suggestions regarding the temporal dynamics underlying this task-order repetition advantage had to remain speculative.

The notion of pre-activating sensory modalities as a function of the previous trial bears a close resemblance to the “Modality-Weighting” Account (MWA; Töllner et al., 2009), originally devised to explain the “modality-shift” effect in cross-modal attention studies (e.g., Spence et al., 2001). According to the MWA, the outcome of pre-attentive saliency computations guides the deployment of focal-attentional selection (analogous to the dimensional-weighting idea of Müller and colleagues for the visual modality; e.g., Gramann et al., 2007, 2010; Müller et al., 2010). In more detail, it is assumed that basic target (e.g., feature contrast) signals computed in separate modality-specific processing modules (e.g., vision and audition) can be top-down weighted prior to being integrated by units of the attention-guiding overall-saliency map. Critically, however, the total amount of available weight is limited, so that an increase in the weight for one modality goes at the expense of the other modality. Following this logic, being able to predict the correct modality (i.e., with predictable task orders) might have led participants to top-down set themselves to the respective sensory modality of the first task (say: vision), leading to an enhanced coding of target signals—as indicated by stronger PCN activations—and, thus, faster selection of targets defined in this weighted (i.e., vision), relative to the non-weighted (in the example, audition), modality. At the same time, this would help participants shield processing for the first task against interference from (second target) signals defined in a sensory modality other than the first target (analogous to dimension-based shielding within a modality; e.g., Müller et al., 2009). This shielding effect would be most prominent under conditions in which the second target signal occurs in close succession to the first target, such as in the present short to intermediate SOA conditions. By contrast, when the task order is non-predictable, weighting of the correct modality is feasible only at chance level, as a result of which a temporally close signal defined in a modality other than the first signal would capture processing resources. However, when the temporal distance between the two targets becomes relatively large—as in the present long SOA condition—there is no longer any interference, so that processing of the (first) visual target can proceed as smoothly with non-predictable as with predictable task orders.

Regarding the *second* task, we again observed the timing (but not the magnitude) of the PCN to be interactively determined by both factors: for predictive task orders, the PCN was (on average ~27 ms) delayed with short inter-task intervals, with a graded decrease in PCN latencies for visual targets presented following intermediate and long intervals after the first (auditory) target. By contrast, no SOA effect was evident for non-predictive task orders. This clearly demonstrates that already early, pre-attentive perceptual encoding processes contribute to the well-established SOA effect of increased behavioral RTs to the second task. Of theoretical significance, this finding indicates that there is a limit to the total amount of attentional resources not only within a given sensory modality, but also across modalities—the central assumption of the MWA (Töllner et al., 2009). Restated, perceptual processing resources are not confined to a single sensory modality, but can be shifted from one modality to another in order to optimize target processing. For the present study, this implies that participants needed more time to focally select the visual target item with short (relative to intermediate and long) inter-task SOAs because a significant amount of cross-modal attentional processing resources was already captured by, and still bound to, the first, auditory target stimulus. At intermediate and longer SOAs, however, these resources became available again, thus expediting focal target selection in the visual modality.

The absence of differences in PCN magnitude across different inter-task intervals appears to be at variance with Jolicœur and colleagues (e.g., Brisson and Jolicoeur, 2007a,b, see also Lien and Croswaite, 2011), who observed an SOA effect under predictable task order conditions. In particular, these authors reported attenuated PCN activations for shorter relative to longer SOAs, which they took to suggest that, at relatively short SOAs, participants could not deploy focal attention to the (second) visual target as efficiently when their *central* attention was still engaged on the (first) auditory target. One reason for the absence of such an SOA effect in the present study might lie in the particular SOA introduced, namely: 150, 300, and 600 ms. By contrast, Brisson and Jolicoeur (2007a) had also used conditions with much longer SOAs (300, 650, and 1000 ms), thus introducing greater differences between the SOAs. Accordingly, any differences among the SOAs used in the present study may have been too small to yield statistically significant effects. In fact, comparing the signal strength of the PCNs to the second task with those elicited by the first task discloses at least a numerical reduction (on average ~0.4 μV) which—in line with Brisson and Jolicoeur (2007a,b)—might reflect the automatic engagement of attentional processing resources on the just previously presented auditory target stimulus.

### Electroencephalographic measurement of motor capacity limitations in dual-task

Evidence for capacity limitations existing at the motor stage of processing is revealed from the activation pattern of the LRP (e.g., Coles, 1989). Recall that, in the present study, all LRP activation values were derived time-locked to the onset of the respective motor response. As demonstrated by Miller (2007), amongst others, the onset latency of this lateralized brain response reflects the time required by the human motor system to activate and execute the motor response, whereas its activation strength indicates how forceful the response produced was. Accordingly, both LRP parameters mirror pure motor processes occurring after the completion of *central* stimulus-response translation (i.e., response selection) processes.

For the *first* task, we found response execution times—as indexed by faster LRP onset latencies—to be greatly expedited (on average ~53 ms) when participants knew, rather than did not know, in advance the order of the two upcoming (dual) tasks. This finding can be explained by the operation of another weighting mechanism: one located at the stage of motor-response production. According to this notion of response weighting (“Response-Weighting” Account, RWA; e.g., Töllner et al., 2012b), participants might top-down set their response system to the respective motor effector (e.g., left index finger) used for the first task in advance when the task order can be correctly anticipated. This, in turn, would lead to the pre-activation of motor units that represent this response (relative to other response alternatives), so that, when the first task is presented, less additional motor evidence would be required to reach the threshold for response initiation and execution. In other words, the RWA assumes a limit to the total amount of processing resources that can be allocated to the various motor responses (i.e., effectors) on the motor production stage. Thus, weighting of one motor effector would lead to facilitated processing of this response, and goes at the expense of alternative motor responses. Note that this notion is different from the MWA (see above), which assumes limited attentional resources that can be allocated to the various sensory modalities (e.g., vision, audition) at a pre-attentive level, affecting the integration of modality-specific feature contrast signals at the attention-guiding master map. As further evidenced by a recent compound-search study (Töllner et al., 2008; Wiegand et al., in press), weighting mechanisms within sensory modalities operate independently of response-based weighting dynamics.

With respect to the *second* task, we observed the timing of the LRP (relative to response onset) to be speeded (by on average ~28 ms) for long, as compared to both intermediate and short, SOAs. This pattern of rLRP onset timing differences replicates the observations of Osman and Moore (1993) who had likewise observed such a numerical (though, in this study, statistically non-significant) pattern of speeded processing times for long relative to both short and intermediate inter-task intervals at the stage of response execution. This demonstrates that motor response dynamics can—together with perceptual and central processing dynamics— contribute to the classic, behavioral SOA effect on RTs to the second task, traditionally assumed to originate exclusively from central bottlenecks. Accordingly, the present findings indicate that the execution of simple (e.g., button press) responses can be affected by the motor system having had to execute another (prior) response at a short time beforehand (as in the present short and intermediate SOA conditions), even when this first response was initiated by the motor cortex of the contralateral hemisphere. By contrast, sufficient time in-between the two dual tasks (as in the present long SOA condition) substantially reduces the time demands required for executing the motor response of the second task. The present electrophysiological evidence of impaired response execution for the second task is also in line with a recent behavioral study by Ulrich et al. (2006), who systematically manipulated the temporal demands for executing the first response. Specifically, participants had to respond to the first task by performing a ballistic movement, namely, move a slider to one of two possible target locations indicated by the pitch of a tone (see Ulrich et al., 2006, for further methodological details). Crucially, Ulrich et al. found that RTs to the second task increased systematically with increasing response execution demands for the first task, which they took to suggest that response execution can be part of the processing bottleneck(s) in classical PRP paradigms.

## Conclusion

**Figure 7:**
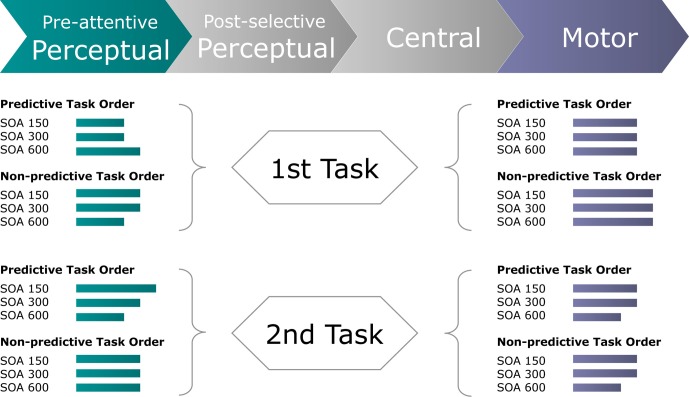
**Schematic of the inferred processing times (green and blue-gray lines) required by pre-attentive perceptual and motor stages of the first and second task to perform a dual task for each experimental (TOP × SOA) condition.** Green lines display the processing times required for the perceptual system to focally select the visual target stimulus, as derived from the timing of the Posterior-Contralateral-Negativity. Blue-gray lines display the processing times required for the motor system to produce the motor response, as derived from the timing of the response-locked Lateralized-Readiness-Potential.

In conclusion, the present study was designed to examine for dual-task interference arising at processing stages either prior or subsequent to central-stage processing. We found TOP to interact with inter-task SOA in determining the speed of (visual) perceptual processes for both the first and the second task. By contrast, response execution times were influenced independently by TOP for the first, and by inter-task SOA for the second, task (see Figure 7). Together, this set of findings complements classical (e.g., Pashler, 1994) as well as advanced versions (e.g., Sigman and Dehaene, 2006) of the central bottleneck model by providing electrophysiological evidence for modulations of both perceptual and motor processing dynamics that, in summation with central capacity limitations, give rise to the well-known behavioral PRP outcome.

### Conflict of interest statement

The authors declare that the research was conducted in the absence of any commercial or financial relationships that could be construed as a potential conflict of interest.
